# Improving the Photoelectric Characteristics of MoS_2_ Thin Films by Doping Rare Earth Element Erbium

**DOI:** 10.1186/s11671-016-1729-6

**Published:** 2016-11-22

**Authors:** Miaofei Meng, Xiying Ma

**Affiliations:** Suzhou University of Science and Technology, Kerui Road No. 1, Gaoxin section, Suzhou, 215011 Jiangsu China

**Keywords:** MoS_2_ film, Er doping, Chemical vapor deposition, Photoelectric characteristics, Light absorption, Photoluminescence

## Abstract

We investigated the surface morphologies, crystal structures, and optical characteristics of rare earth element erbium (Er)-doped MoS_2_ (Er: MoS_2_) thin films fabricated on Si substrates via chemical vapor deposition (CVD). The surface mopography, crystalline structure, light absorption property, and the photoelectronic characteristics of the Er: MoS_2_ films were studied. The results indicate that doping makes the crystallinity of MoS_2_ films better than that of the undoped film. Meanwhile, the electron mobility and conductivity of the Er-doped MoS_2_ films increase about one order of magnitude, and the current-voltage (*I*-*V*) and the photoelectric response characteristics of the Er:MoS_2_/Si heterojunction increase significantly. Moreover, Er-doped MoS_2_ films exhibit strong light absorption and photoluminescence in the visible light range at room temperature; the intensity is enhanced by about twice that of the undoped film. The results indicate that the doping of MoS_2_ with Er can significantly improve the photoelectric characteristics and can be used to fabricate highly efficient luminescence and optoelectronic devices.

## Background

Layered quasi-two dimensional (2D) chalcogenide materials have attracted great interest due to their excellent optical, electrical, catalysis, and lubrication characteristics [1–3]. Especially, 2D molybdenum disulfide (MoS_2_) has been widely studied and applied in field-effect transistors [4, 5] and energy harvesting [6, 7]. It has been found that a monolayer of MoS_2_ has a direct bandgap of 1.8 eV when it is stripped from a bulk material that has an indirect bandgap of 1.29 eV [8, 9]. This large change in the energy band holds great potential for applications of MoS_2_ in optoelectronic fields, such as red photodiodes and photodetectors [10, 11]. However, the efficiency of photoluminescence (PL) and photoelectric conversion of 2D MoS_2_ [12] are relatively low. Researchers have explored many avenues to improve the PL intensity and the response rate of MoS_2_ films. For example, Singha et al. found that gold nanoparticles may impose an obvious p-doping effect in single-layer and bi-layer MoS_2_ samples, resulting in enhanced PL [13].

Rare earth elements (REE) are active elements that have been widely added in optoelectronic devices to improve PL and photoelectric conversion efficiency [14, 15]. So far, there have been few reports for REE-doped MoS_2_. Herein, we report a study of the doping effects of rare earth element Er on the surface morphologies, crystal structures, and optical characteristics of MoS_2_ thin films. Pure MoS_2_ and Er: MoS_2_ samples were fabricated on Si substrates by chemical vapor deposition (CVD). Additionally, we systematically analyzed the surface morphologies, structures, and optical absorption characteristics of the samples.

## Methods

Er (NO_3_)_3_·5H_2_O (99.9%) and MoS_2_ powder (AR, 99%) reagents were used as the precursor materials. A mixed solution comprising 1-g analytical grade MoS_2_ micro powder, 1-g analytical grade erbium nitrate pentahydrate (Er(NO_3_)_3_·5H_2_O) crystals, and 200 mL of diluted sulfuric acid (H_2_SO_4_) was formed by mixing the above mentioned components for 5 min, followed which the solution was maintained at 70°C via a water bath. The CVD system consisted of a horizontal quartz tube furnace, a vacuum system, an intake system, and a water bath. The Si substrates were placed in the center of the furnace, and subsequently, the pressure in the furnace was reduced to 10^−2^ Pa and the furnace was heated up to 650°C for 20 min. Ar gas was introduced into the mixed solution at a flow rate of 25 sccm, carrying Er^3+^ and MoS_2_ molecules into the furnace. Furthermore, to investigate the material properties of MoS_2_ films, some pure MoS_2_ samples were deposited on the Si substrates by the same method.

The surface morphologies and crystalline structures of the thin films were characterized using atomic force microscope (AFM) and X-ray diffraction (XRD). The electrical properties of the thin films were analyzed by a Hall Effect Measurement System (HMS-3000, Ecopia, Anyang, South Korea). The ultraviolet-visible (UV-vis) absorption spectra and photoluminescence properties of the samples were investigated by a UV-vis spectrophotometer (Shimadzu UV-3600) and fluorescence spectrophotometer at room temperature. Photocurrent current-voltage (*I*-*V*) curves of the doped and undoped MoS_2_/Si heterojunction were investigated by a semiconductor analysis system (Keithley 4200).

## Results and Discussion

**Fig. 1:**
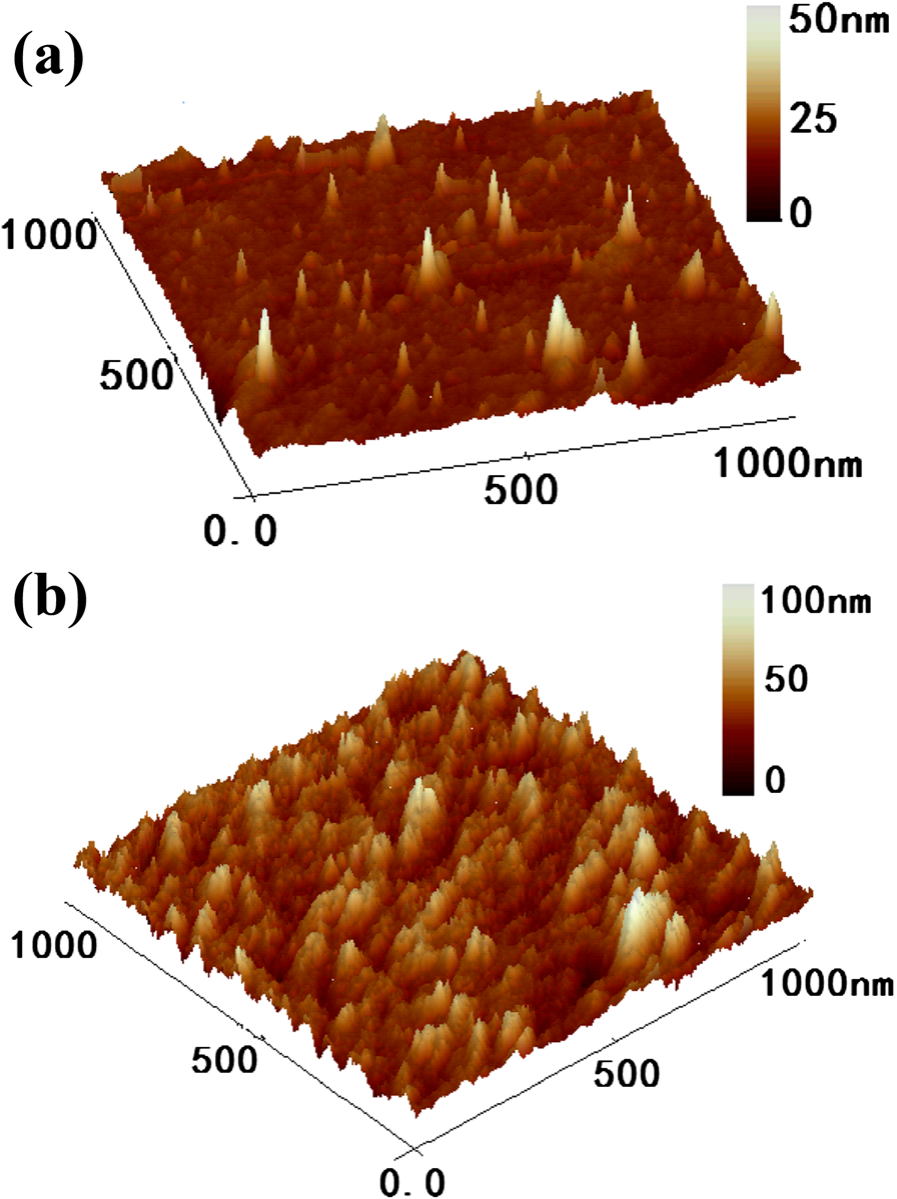
AFM images of MoS_2_ samples. **a** The MoS_2_ film. **b** The Er:MoS_2_ film

The AFM images of the pure MoS_2_ and Er: MoS_2_ thin films on the Si substrates are shown in Fig. 1. The surface of the pure MoS_2_ film in Fig. 1a is a continuous film with an average thickness about 25 nm, and some quantum dots around 20 nm are uniformly scattered on the Si substrate. The Er: MoS_2_ film shown in Fig. 1b is a large fluctuation film composed of compact quantum dots with a uniform color, and the average thickness is about 50 nm. For the same deposition conditions and time, the density and size of the quantum dots in Er: MoS_2_ film increase remarkably resulting from the catalytic action of Er^3+^ on the deposition course.

**Fig. 2:**
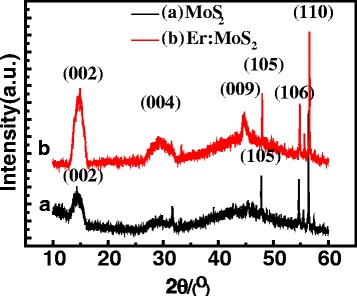
XRD patterns of the MoS_2_ film and the Er:MoS_2_ film for the diffraction angle in the range of 10°~60°

The crystal structures of the synthesized samples were characterized by using the X-ray diffraction (XRD) technique, as shown in Fig. 2. For the pure MoS_2_ sample, there are four sharp diffraction peaks located at 14.7°, 47.8°, 54.6°, and 56.4°, corresponding to the (002), (105), (106), and (110) crystal planes of MoS_2_, respectively, showing that the film is characterized by a polycrystal structure. In the Er: MoS_2_ film, the position of the above four diffraction peaks is almost the same as that of pure MoS_2_. Besides, there are two more peaks at 29.5° and 44.8°, corresponding to the (004) and (009) planes, respectively. No diffraction peaks from elemental Er is observed, indicating that the Er doping does not change the crystal structure of the MoS_2_ film. Er atoms were doped in MoS_2_ film in the way of substitution doping, and Mo atom was replaced by Er element. By doping, the diffraction peaks of MoS_2_ crystal increased and the diffraction intensity was enhanced, showing that doping improved the crystallinity of the MoS_2_ films.

**Fig. 3:**
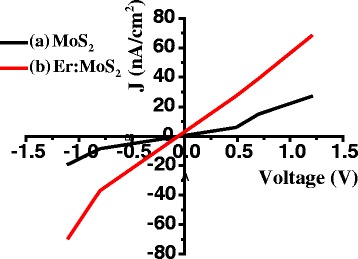
The surface *J*-*V* characteristic curves of the MoS_2_ film and the Er:MoS_2_ film

The surface *J*-*V* properties, carrier mobilities, and Hall coefficients of the MoS_2_ and Er: MoS_2_ samples were measured using a Hall Effect measurement system via the four measured points on the samples at dark condition, as shown in Fig. 3. The currents of the samples show a linear dependency on the applied voltage, revealing that the films have a good conductivity. The slopes of the *J*-*V* curves show the resistivity of the MoS_2_ samples. The curve of the Er: MoS_2_ film has good linearity and a small slope, with the films showing a significant reduction in resistivity when Er ions are doped. According to the equation for calculation of mobility: *σ* = *nqμ* (*σ* is conductivity, *n* is electron concentration, *q* is electron charge, *μ* is mobility), the electron motilities in the MoS_2_ and Er: MoS_2_ films are 3.996 × 10^3^ cm^2^/Vs and 5.547 × 10^3^ cm^2^/Vs, respectively. Note that the mobility value for the MoS_2_ film is obviously improved by doping Er^3+^. Furthermore, According to the equation for the Hall coefficients: *ε*_y_ = *R*_H_ *J*_x_ *B*_z_ (*ε*_y_ is electric field intensity, *R*_H_ is Hall coefficients, *J*_x_ is current density, *B*_z_ is magnetic induction intensity), the Hall coefficients of the MoS_2_ and Er: MoS_2_ films are 1.905 × 10^7^ cm^3^/C and 4.581 × 10^8^ cm^3^/C, respectively, showing that the films are *p*-type semiconductors. The *J*-*V* curves in the MoS_2_ film show a significant decrease in resistivity after Er doping. Good conductive properties can reduce the surface heat loss in the photodetector, thereby increasing the lifetime and frequency response of the MoS_2_ photovoltaic device.

**Fig. 4:**
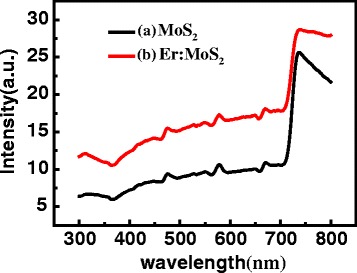
The absorption spectra of the MoS_2_ film and the Er:MoS_2_ film

Figure 4 shows the absorption spectra of the pure MoS_2_ and Er: MoS_2_ films in the visible light range. Clearly, the absorption of the Er: MoS_2_ film is enhanced significantly by doping Er, attributing to the absorptions of Er ions and the impurity energy level in the bandgap of MoS_2_ by doping Er. Additionally, a few maximum values emerge at 475, 578, 670, and 735 nm in the absorption spectra, showing that the film has strong light absorption in these wavebands. The absorption peak at 670 nm corresponds to a bandgap width of 1.85 eV in the MoS_2_ film, close to the energy gap of a monolayer of MoS_2,_ 1.80 eV. MoS_2_ films have strong absorption at 735 nm, which can be considered as the optical absorption edge, corresponding to a bandgap width of 1.69 eV. Therefore, the doping of Er significantly improves the light absorption and does not change the position of the absorption peak. The increase of the light absorption of Er^3+^-doped MoS_2_ film can be improved by the photoelectric transformation and photovoltaic effect of MoS_2_ semiconductor devices.

**Fig. 5:**
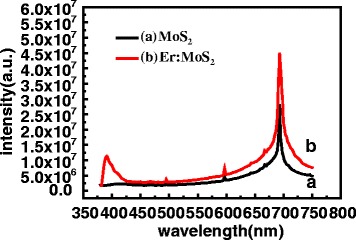
The photoluminescence spectra of the MoS_2_ film and the Er:MoS_2_ film

Figure 5 shows the photoluminescence spectra of the MoS_2_ and Er: MoS_2_ film excited by 360 nm light at room temperature. In the pure MoS_2_ film, an obvious PL peak is centered at 693 nm, coinciding with the intrinsic radiative transition photoluminescence of the single-layer MoS_2_. In the Er: MoS_2_ film, two significantly enhanced PL peaks are located at 394 and 693 nm each. The peak at 394 nm is due to the transitions from the ^2^H_11/2_ energy level to the ground state ^4^I_15/2_ [16–18]. The intense peak at 693 nm is largely enhanced result from the direct-gap luminescence of MoS_2_. It is important to note that the PL intensity of the Er: MoS_2_ film is almost twice as strong as that of the undoped film, i.e., the doping of Er in MoS_2_ can largely improve the absorption and photoluminescence efficiency of MoS_2_, which in turn acts as an exciting active center in the film.

**Fig. 6:**
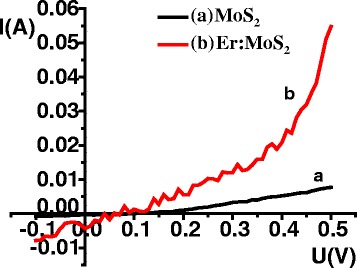
Photocurrent *I*-*V* curves of the doped and undoped MoS_2_/Si heterojunction

The photocurrent *I*-*V* behavior of the MoS_2_-Si heterojunction was obtained while irradiating the surface of the films by a standard white light with a power of 100 mW/cm^2^, as shown in Fig. 6. For two samples, the current increases exponentially with an increase in the voltage. The short-circuit currents (*I*_SC_) of the MoS_2_ and Er: MoS_2_ film samples are 0.392 and 4.35 mA, respectively, and the open-circuit voltage (*U*_OC_) is 49.98 and 90.02 mV, respectively. Obviously, after Er doping the short-circuit current and open-circuit voltage both increase significantly. This is because the doped Er ions will increase light absorption, resulting in an increase in the number of photo-generated carriers and finally enhancing the photocurrent response.

## Conclusions

We have studied the effects of Er doping on the surface morphologies, crystalline, optical absorption, PL, and photoelectrical properties of MoS_2_ films. We found that the Er^3+^ ions do not change the crystal structure of MoS_2_ films but make the crystallinity better. At the same time, Er^3+^ doping improves the carrier mobility and enhances the current-voltage (*I*-*V*) characteristics of the MoS_2_ thin films. Additionally, Er^3+^-doped MoS_2_ films exhibit stronger light absorption and photoluminescence in the visible light range at room temperature. The results show that Er^3+^-doped MoS_2_ film can be used to fabricate highly efficient luminescence and optoelectronic devices.

## Abbreviations

2D: Quasi-two dimensional
AFM: Atomic force microscope
CVD: Chemical vapor deposition
Er(NO_3_)_3_·5H_2_O: Erbium nitrate pentahydrate
Er: Erbium
Er: MoS_2_: Erbium-doped MoS_2_
H_2_SO_4_: Sulfuric acid
*I*_SC_: Short-circuit currents
*I*-*V*: Current-voltage
PL: Photoluminescence
REE: Rare earth elements
*U*_OC_: Open-circuit voltage
UV-vis: Ultraviolet-visible
XRD: X-ray diffraction
